# 2-Methyl-3,5,6-triphenyl-2,3-dihydro­pyrazine

**DOI:** 10.1107/S1600536809005042

**Published:** 2009-02-18

**Authors:** N. Anuradha, A. Thiruvalluvar, K. Pandiarajan, S. Chitra, R. J. Butcher

**Affiliations:** a Postgraduate Research Department of Physics, Rajah Serfoji Government College (Autonomous), Thanjavur 613 005, Tamil Nadu, India; b Department of Chemistry, Annamalai University, Annamalai Nagar 608 002, Tamil Nadu, India; c Department of Chemistry, Howard University, 525 College Street NW, Washington, DC 20059, USA

## Abstract

In the title mol­ecule, C_23_H_20_N_2_, the heterocyclic ring adopts a screw-boat conformation, with all substituents equatorial. The phenyl ring at position 3 makes dihedral angles of 78.12 (15) and 72.67 (15)°, respectively, with the phenyl rings at positions 5 and 6; the dihedral angle between the phenyl rings at positions 5 and 6 is 67.32 (14)°. A C—H⋯π inter­action is present in the crystal structure.

## Related literature

Some 2-alkyl-3,5,6-triphenyl-2,3-dihydro­pyrazines have been reported to exhibit fluorescence in the solid-state, see: Baliah & Pandiarajan (1978 ▶). For the use of dihydro­pyrazines in medicine, in particular with reference to DNA breakage activity, see: Yamaguchi *et al.* (2003 ▶). For their biological activity, see: Takechi *et al.* (2004 ▶) and their cyclo­oxygenase inhibitory activity, see: Singh *et al.* (2004 ▶).
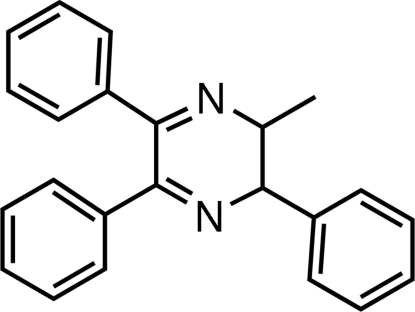


## Experimental

### 

#### Crystal data

C_23_H_20_N_2_
*M* *_r_* = 324.41
Triclinic, 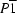

*a* = 10.4406 (10) Å
*b* = 10.5753 (7) Å
*c* = 11.0810 (13) Å
α = 93.439 (8)°
β = 114.161 (10)°
γ = 118.343 (9)°
*V* = 931.9 (2) Å^3^
*Z* = 2
Cu *K*α radiation
μ = 0.52 mm^−1^
*T* = 295 K
0.41 × 0.36 × 0.28 mm

#### Data collection

Oxford Diffraction Gemini R diffractometer
Absorption correction: analytical (*CrysAlis RED*; Oxford Diffraction, 2008 ▶) *T*_min_ = 0.831, *T*_max_ = 0.885
8513 measured reflections
3852 independent reflections
3195 reflections with *I* > 2σ(*I*)
*R*_int_ = 0.045

#### Refinement

*R*[*F* ^2^ > 2σ(*F* ^2^)] = 0.086
*wR*(*F* ^2^) = 0.270
*S* = 1.09
3852 reflections
227 parameters
H-atom parameters constrained
Δρ_max_ = 0.47 e Å^−3^
Δρ_min_ = −0.27 e Å^−3^

### 

Data collection: *CrysAlis CCD* (Oxford Diffraction, 2008 ▶); cell refinement: *CrysAlis RED* (Oxford Diffraction, 2008 ▶); data reduction: *CrysAlis RED*; program(s) used to solve structure: *SIR2004* (Burla *et al.*, 2005 ▶); program(s) used to refine structure: *SHELXL97* (Sheldrick, 2008 ▶); molecular graphics: *ORTEP-3* (Farrugia, 1997 ▶); software used to prepare material for publication: *PLATON* (Spek, 2009 ▶).

## Supplementary Material

Crystal structure: contains datablocks global, I. DOI: 10.1107/S1600536809005042/tk2373sup1.cif

Structure factors: contains datablocks I. DOI: 10.1107/S1600536809005042/tk2373Isup2.hkl

Additional supplementary materials:  crystallographic information; 3D view; checkCIF report

## Figures and Tables

**Table 1:** Hydrogen-bond geometry (Å, °) *Cg* is the centroid of the C31–C36 ring.

*D*—H⋯*A*	*D*—H	H⋯*A*	*D*⋯*A*	*D*—H⋯*A*
C65—H65⋯*Cg*^i^	0.93	2.97	3.834 (3)	156

Symmetry code: (i) 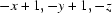.

**Fig. 1.**
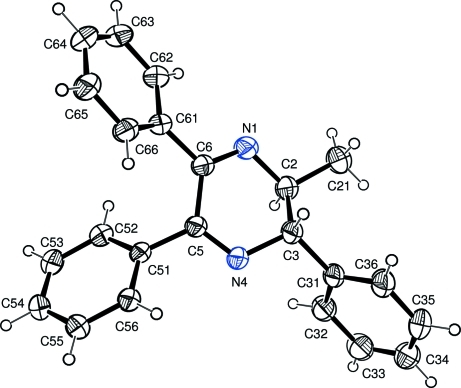
The molecular structure of (I), showing the atom-numbering scheme and displacement ellipsoids drawn at the 30% probability level. H atoms are shown as small spheres of arbitrary radius.

## supplementary crystallographic information

### Comment

Some 2-alkyl-3,5,6-triphenyl-2,3-dihydropyrazines have been reported to exhibit fluorescence in the solid-state (Baliah & Pandiarajan, 1978). Dihydropyrazines are used in medicine, in particular with reference to DNA breakage activity (Yamaguchi *et al.*, 2003). They also exhibit biological effects (Takechi *et al.*, 2004) such as growth inhibition of *Escherichia coli* and cyclooxygenase inhibitory activity (Singh *et al.*, 2004).

In the title molecule (I), Fig. 1, the heterocyclic ring adopts a screw-boat conformation, with all substituents equatorial. The phenyl ring at position 3 forms dihedral angles of 78.12 (15)° and 72.67 (15)° with the phenyl rings at positions 5 and 6, respectively. The dihedral angle between the phenyl rings at positions 5 and 6 is 67.32 (14)°. The most prominent intermolecular interaction in the crystal structure is a C65—H65···π contact involving the C31–C36 phenyl ring (Table 1).

### Experimental

To a homogeneous solution of benzil (1.05 g, 0.005 mol) and 1-methyl-2-phenyl-1,2-ethanediamine dihydrochloride (1.11 g, 0.005 mol) in ethanol (20 ml), sodium acetate trihydrate (2.04 g, 0.015 mol) was added. The precipitated sodium chloride was filtered off and the filtrate was refluxed for 2 h. On completion of the reaction, as indicated by TLC, the reaction mixture was poured into crushed ice and the resulting solid was filtered and purified by column chromatography on silica gel. Elution with benzene–petroleum ether 333–353 K (4:1 v/v) gave the pure product in 1.72 g (80%) yield. .

### Refinement

H atoms were positioned geometrically and allowed to ride on their parent atom with C—H = 0.93 to 0.98 Å, and with *U*_iso_(H) = 1.2 to 1.5 times *U*_eq_(C).

### Crystal data

**Table:**

C_23_H_20_N_2_	*Z* = 2
*M**_r_* = 324.41	*F*(000) = 344
Triclinic, *P*1	*D*_x_ = 1.156 Mg m^−^^3^
Hall symbol: -P 1	Melting point: 460 K
*a* = 10.4406 (10) Å	Cu *K*α radiation, λ = 1.54184 Å
*b* = 10.5753 (7) Å	Cell parameters from 5680 reflections
*c* = 11.0810 (13) Å	θ = 5.2–77.4°
α = 93.439 (8)°	µ = 0.52 mm^−^^1^
β = 114.161 (10)°	*T* = 295 K
γ = 118.343 (9)°	Prism, pale yellow
*V* = 931.9 (2) Å^3^	0.41 × 0.36 × 0.28 mm

### Data collection

**Table:**

Oxford Diffraction Gemini R diffractometer	3852 independent reflections
Radiation source: fine-focus sealed tube	3195 reflections with *I* > 2σ(*I*)
graphite	*R*_int_ = 0.045
Detector resolution: 10.5081 pixels mm^-1^	θ_max_ = 77.5°, θ_min_ = 5.2°
φ and ω scans	*h* = −13→13
Absorption correction: analytical (*CrysAlis RED*; Oxford Diffraction, 2008)	*k* = −13→10
*T*_min_ = 0.831, *T*_max_ = 0.885	*l* = −13→13
8513 measured reflections	

### Refinement

**Table:**

Refinement on *F*^2^	Primary atom site location: structure-invariant direct methods
Least-squares matrix: full	Secondary atom site location: difference Fourier map
*R*[*F*^2^ > 2σ(*F*^2^)] = 0.086	Hydrogen site location: inferred from neighbouring sites
*wR*(*F*^2^) = 0.270	H-atom parameters constrained
*S* = 1.09	*w* = 1/[σ^2^(*F*_o_^2^) + (0.1784*P*)^2^ + 0.1376*P*] where *P* = (*F*_o_^2^ + 2*F*_c_^2^)/3
3852 reflections	(Δ/σ)_max_ = 0.001
227 parameters	Δρ_max_ = 0.47 e Å^−^^3^
0 restraints	Δρ_min_ = −0.27 e Å^−^^3^

### Special details

**Table:**

Geometry. Bond distances, angles *etc*. have been calculated using the rounded fractional coordinates. All su's are estimated from the variances of the (full) variance-covariance matrix. The cell e.s.d.'s are taken into account in the estimation of distances, angles and torsion angles
Refinement. Refinement of *F*^2^ against ALL reflections. The weighted *R*-factor *wR* and goodness of fit *S* are based on *F*^2^, conventional *R*-factors *R* are based on *F*, with *F* set to zero for negative *F*^2^. The threshold expression of *F*^2^ > 2σ(*F*^2^) is used only for calculating *R*-factors(gt) *etc*. and is not relevant to the choice of reflections for refinement. *R*-factors based on *F*^2^ are statistically about twice as large as those based on *F*, and *R*- factors based on ALL data will be even larger.

### Fractional atomic coordinates and isotropic or equivalent isotropic displacement parameters (Å^2^)

**Table:**

	*x*	*y*	*z*	*U*_iso_*/*U*_eq_	
N1	0.0780 (2)	0.1850 (2)	−0.0471 (2)	0.0633 (6)	
N4	0.39318 (19)	0.25469 (17)	−0.01087 (17)	0.0512 (5)	
C2	0.0900 (3)	0.0714 (2)	−0.1158 (3)	0.0613 (7)	
C3	0.2375 (3)	0.1513 (2)	−0.1432 (2)	0.0551 (6)	
C5	0.3812 (2)	0.3247 (2)	0.0777 (2)	0.0491 (5)	
C6	0.2163 (2)	0.3057 (2)	0.0441 (2)	0.0536 (6)	
C21	−0.0774 (3)	−0.0383 (3)	−0.2471 (3)	0.0824 (9)	
C31	0.2605 (3)	0.0442 (2)	−0.2156 (2)	0.0587 (6)	
C32	0.3089 (3)	−0.0438 (3)	−0.1466 (3)	0.0712 (8)	
C33	0.3318 (4)	−0.1415 (3)	−0.2108 (3)	0.0873 (10)	
C34	0.3090 (4)	−0.1517 (3)	−0.3418 (3)	0.0930 (10)	
C35	0.2604 (4)	−0.0662 (4)	−0.4115 (3)	0.0901 (10)	
C36	0.2364 (3)	0.0324 (3)	−0.3491 (3)	0.0732 (8)	
C51	0.5327 (2)	0.4176 (2)	0.2180 (2)	0.0498 (6)	
C52	0.5222 (3)	0.4188 (3)	0.3387 (3)	0.0634 (7)	
C53	0.6663 (3)	0.4975 (3)	0.4688 (3)	0.0739 (9)	
C54	0.8218 (3)	0.5780 (3)	0.4792 (3)	0.0725 (8)	
C55	0.8337 (3)	0.5780 (3)	0.3593 (3)	0.0658 (7)	
C56	0.6909 (2)	0.4972 (2)	0.2294 (2)	0.0539 (6)	
C61	0.2079 (3)	0.4259 (2)	0.1105 (2)	0.0537 (6)	
C62	0.0694 (3)	0.3844 (3)	0.1264 (3)	0.0638 (7)	
C63	0.0531 (3)	0.4931 (3)	0.1805 (3)	0.0762 (10)	
C64	0.1712 (4)	0.6439 (3)	0.2153 (3)	0.0806 (10)	
C65	0.3098 (3)	0.6872 (3)	0.1991 (3)	0.0750 (9)	
C66	0.3283 (3)	0.5782 (3)	0.1468 (3)	0.0630 (7)	
H2	0.11272	0.01451	−0.05249	0.0736*	
H3	0.21696	0.21204	−0.20281	0.0661*	
H21A	−0.16525	−0.07906	−0.22411	0.1236*	
H21B	−0.07434	−0.11930	−0.28702	0.1236*	
H21C	−0.09824	0.01403	−0.31308	0.1236*	
H32	0.32603	−0.03712	−0.05663	0.0854*	
H33	0.36313	−0.20078	−0.16377	0.1046*	
H34	0.32629	−0.21646	−0.38372	0.1115*	
H35	0.24329	−0.07406	−0.50159	0.1078*	
H36	0.20407	0.09047	−0.39721	0.0879*	
H52	0.41748	0.36626	0.33246	0.0761*	
H53	0.65783	0.49590	0.54911	0.0886*	
H54	0.91824	0.63205	0.56637	0.0869*	
H55	0.93864	0.63279	0.36618	0.0790*	
H56	0.70022	0.49594	0.14949	0.0647*	
H62	−0.01299	0.28242	0.10022	0.0765*	
H63	−0.03835	0.46414	0.19338	0.0914*	
H64	0.15848	0.71699	0.24976	0.0967*	
H65	0.39029	0.78941	0.22342	0.0900*	
H66	0.42124	0.60729	0.13614	0.0757*	

### Atomic displacement parameters (Å^2^)

**Table:**

	*U*^11^	*U*^22^	*U*^33^	*U*^12^	*U*^13^	*U*^23^
N1	0.0477 (9)	0.0567 (10)	0.0707 (12)	0.0264 (8)	0.0224 (8)	0.0037 (8)
N4	0.0459 (8)	0.0449 (8)	0.0534 (9)	0.0233 (7)	0.0207 (7)	0.0036 (6)
C2	0.0515 (11)	0.0536 (11)	0.0620 (12)	0.0248 (9)	0.0216 (9)	0.0032 (9)
C3	0.0529 (10)	0.0477 (10)	0.0524 (11)	0.0283 (8)	0.0171 (9)	0.0030 (8)
C5	0.0431 (9)	0.0440 (9)	0.0542 (10)	0.0234 (7)	0.0211 (8)	0.0071 (7)
C6	0.0453 (9)	0.0514 (10)	0.0587 (11)	0.0266 (8)	0.0229 (8)	0.0072 (8)
C21	0.0577 (13)	0.0709 (15)	0.0799 (17)	0.0244 (11)	0.0199 (12)	−0.0044 (12)
C31	0.0504 (10)	0.0485 (10)	0.0551 (11)	0.0260 (8)	0.0127 (8)	−0.0046 (8)
C32	0.0698 (14)	0.0611 (12)	0.0694 (14)	0.0411 (11)	0.0196 (11)	0.0067 (10)
C33	0.0798 (17)	0.0651 (14)	0.092 (2)	0.0482 (13)	0.0160 (14)	−0.0018 (13)
C34	0.0795 (17)	0.0765 (17)	0.093 (2)	0.0488 (14)	0.0179 (15)	−0.0205 (15)
C35	0.0881 (19)	0.094 (2)	0.0633 (15)	0.0484 (16)	0.0244 (14)	−0.0110 (14)
C36	0.0730 (14)	0.0702 (14)	0.0593 (13)	0.0419 (12)	0.0185 (11)	0.0014 (10)
C51	0.0462 (9)	0.0463 (9)	0.0546 (11)	0.0275 (8)	0.0221 (8)	0.0048 (7)
C52	0.0570 (11)	0.0717 (13)	0.0623 (12)	0.0368 (10)	0.0303 (10)	0.0081 (10)
C53	0.0755 (15)	0.0947 (17)	0.0516 (12)	0.0510 (14)	0.0290 (11)	0.0056 (11)
C54	0.0594 (12)	0.0782 (15)	0.0551 (12)	0.0384 (11)	0.0115 (10)	−0.0064 (10)
C55	0.0464 (10)	0.0626 (12)	0.0670 (13)	0.0249 (9)	0.0197 (10)	−0.0003 (10)
C56	0.0481 (10)	0.0512 (10)	0.0552 (11)	0.0256 (8)	0.0233 (8)	0.0057 (8)
C61	0.0484 (10)	0.0577 (10)	0.0552 (11)	0.0330 (9)	0.0224 (8)	0.0101 (8)
C62	0.0544 (11)	0.0722 (13)	0.0677 (13)	0.0380 (10)	0.0296 (10)	0.0164 (10)
C63	0.0703 (14)	0.1005 (19)	0.0767 (16)	0.0583 (15)	0.0393 (12)	0.0184 (14)
C64	0.0844 (17)	0.0880 (17)	0.0815 (17)	0.0620 (15)	0.0363 (14)	0.0059 (13)
C65	0.0728 (14)	0.0619 (13)	0.0844 (17)	0.0404 (12)	0.0326 (13)	0.0053 (11)
C66	0.0554 (11)	0.0586 (12)	0.0741 (14)	0.0331 (10)	0.0309 (10)	0.0084 (10)

### Geometric parameters (Å, °)

**Table:**

N1—C2	1.466 (4)	C62—C63	1.377 (5)
N1—C6	1.279 (3)	C63—C64	1.372 (4)
N4—C3	1.469 (3)	C64—C65	1.389 (6)
N4—C5	1.276 (3)	C65—C66	1.387 (5)
C2—C3	1.535 (4)	C2—H2	0.9800
C2—C21	1.519 (4)	C3—H3	0.9800
C3—C31	1.514 (4)	C21—H21A	0.9600
C5—C6	1.510 (3)	C21—H21B	0.9600
C5—C51	1.487 (3)	C21—H21C	0.9600
C6—C61	1.487 (3)	C32—H32	0.9300
C31—C32	1.386 (4)	C33—H33	0.9300
C31—C36	1.382 (4)	C34—H34	0.9300
C32—C33	1.384 (5)	C35—H35	0.9300
C33—C34	1.360 (4)	C36—H36	0.9300
C34—C35	1.368 (5)	C52—H52	0.9300
C35—C36	1.390 (5)	C53—H53	0.9300
C51—C52	1.386 (4)	C54—H54	0.9300
C51—C56	1.396 (3)	C55—H55	0.9300
C52—C53	1.388 (4)	C56—H56	0.9300
C53—C54	1.377 (5)	C62—H62	0.9300
C54—C55	1.384 (4)	C63—H63	0.9300
C55—C56	1.382 (4)	C64—H64	0.9300
C61—C62	1.389 (5)	C65—H65	0.9300
C61—C66	1.386 (4)	C66—H66	0.9300
			
N1···N4	2.848 (3)	H2···C5	2.8400
N4···N1	2.848 (3)	H2···C32	2.9200
N1···H62	2.5700	H2···H32	2.5500
N1···H2^i^	2.7800	H2···N1^i^	2.7800
N4···H32	2.7800	H3···C6	2.8600
N4···H56	2.6400	H3···H21C	2.5600
N4···H33^ii^	2.8300	H3···H36	2.3600
N4···H66^iii^	2.7500	H3···C55^iii^	3.0100
C32···C32^ii^	3.585 (5)	H3···C56^iii^	2.8300
C51···C66	3.207 (4)	H21B···C31	2.7700
C52···C66	3.407 (5)	H21C···H3	2.5600
C52···C61	3.261 (4)	H21C···C35^v^	3.0600
C61···C52	3.261 (4)	H21C···H35^v^	2.3300
C62···C64^iv^	3.479 (4)	H32···N4	2.7800
C64···C62^iv^	3.479 (4)	H32···C2	3.0500
C66···C51	3.207 (4)	H32···H2	2.5500
C66···C52	3.407 (5)	H32···C33^ii^	3.0600
C2···H32	3.0500	H33···N4^ii^	2.8300
C5···H2	2.8400	H33···C51^ii^	3.0900
C5···H66	2.8100	H33···C56^ii^	2.9000
C6···H52	2.8300	H35···H21C^v^	2.3300
C6···H3	2.8600	H36···H3	2.3600
C31···H21B	2.7700	H52···C6	2.8300
C32···H2	2.9200	H52···C61	2.8900
C33···H32^ii^	3.0600	H52···H53^vi^	2.4800
C35···H65^iii^	3.0200	H53···C52^vi^	3.0500
C35···H21C^v^	3.0600	H53···H52^vi^	2.4800
C36···H65^iii^	2.9900	H56···N4	2.6400
C51···H66	2.7900	H62···N1	2.5700
C51···H33^ii^	3.0900	H65···C35^iii^	3.0200
C52···H53^vi^	3.0500	H65···C36^iii^	2.9900
C55···H3^iii^	3.0100	H66···C5	2.8100
C56···H33^ii^	2.9000	H66···C51	2.7900
C56···H3^iii^	2.8300	H66···N4^iii^	2.7500
C61···H52	2.8900		
			
C2—N1—C6	116.8 (2)	C21—C2—H2	108.00
C3—N4—C5	116.3 (2)	N4—C3—H3	108.00
N1—C2—C3	109.45 (18)	C2—C3—H3	108.00
N1—C2—C21	109.0 (3)	C31—C3—H3	108.00
C3—C2—C21	113.5 (2)	C2—C21—H21A	109.00
N4—C3—C2	109.53 (19)	C2—C21—H21B	109.00
N4—C3—C31	109.3 (2)	C2—C21—H21C	109.00
C2—C3—C31	113.97 (18)	H21A—C21—H21B	109.00
N4—C5—C6	121.10 (19)	H21A—C21—H21C	110.00
N4—C5—C51	117.5 (2)	H21B—C21—H21C	109.00
C6—C5—C51	121.34 (19)	C31—C32—H32	120.00
N1—C6—C5	120.0 (2)	C33—C32—H32	120.00
N1—C6—C61	117.9 (2)	C32—C33—H33	120.00
C5—C6—C61	122.14 (19)	C34—C33—H33	120.00
C3—C31—C32	119.6 (2)	C33—C34—H34	120.00
C3—C31—C36	121.8 (2)	C35—C34—H34	120.00
C32—C31—C36	118.5 (3)	C34—C35—H35	120.00
C31—C32—C33	120.4 (3)	C36—C35—H35	120.00
C32—C33—C34	120.7 (3)	C31—C36—H36	120.00
C33—C34—C35	119.6 (4)	C35—C36—H36	120.00
C34—C35—C36	120.7 (3)	C51—C52—H52	120.00
C31—C36—C35	120.1 (3)	C53—C52—H52	120.00
C5—C51—C52	121.7 (2)	C52—C53—H53	120.00
C5—C51—C56	119.25 (19)	C54—C53—H53	120.00
C52—C51—C56	118.9 (2)	C53—C54—H54	120.00
C51—C52—C53	120.7 (3)	C55—C54—H54	120.00
C52—C53—C54	120.1 (3)	C54—C55—H55	120.00
C53—C54—C55	119.7 (3)	C56—C55—H55	120.00
C54—C55—C56	120.5 (3)	C51—C56—H56	120.00
C51—C56—C55	120.1 (2)	C55—C56—H56	120.00
C6—C61—C62	119.0 (2)	C61—C62—H62	120.00
C6—C61—C66	121.5 (3)	C63—C62—H62	120.00
C62—C61—C66	119.4 (3)	C62—C63—H63	120.00
C61—C62—C63	120.5 (3)	C64—C63—H63	120.00
C62—C63—C64	120.2 (4)	C63—C64—H64	120.00
C63—C64—C65	119.9 (3)	C65—C64—H64	120.00
C64—C65—C66	120.1 (3)	C64—C65—H65	120.00
C61—C66—C65	119.8 (3)	C66—C65—H65	120.00
N1—C2—H2	108.00	C61—C66—H66	120.00
C3—C2—H2	108.00	C65—C66—H66	120.00
			
C6—N1—C2—C3	−38.6 (3)	C5—C6—C61—C62	−151.9 (2)
C6—N1—C2—C21	−163.2 (2)	C5—C6—C61—C66	33.1 (3)
C2—N1—C6—C5	−0.8 (3)	C3—C31—C32—C33	−179.5 (3)
C2—N1—C6—C61	177.16 (19)	C36—C31—C32—C33	−0.2 (5)
C5—N4—C3—C2	−37.2 (3)	C3—C31—C36—C35	179.4 (3)
C5—N4—C3—C31	−162.71 (18)	C32—C31—C36—C35	0.1 (5)
C3—N4—C5—C6	−2.4 (3)	C31—C32—C33—C34	0.7 (5)
C3—N4—C5—C51	173.76 (17)	C32—C33—C34—C35	−1.0 (6)
N1—C2—C3—N4	57.9 (3)	C33—C34—C35—C36	0.9 (6)
N1—C2—C3—C31	−179.37 (19)	C34—C35—C36—C31	−0.4 (6)
C21—C2—C3—N4	179.9 (2)	C5—C51—C52—C53	175.2 (3)
C21—C2—C3—C31	−57.3 (3)	C56—C51—C52—C53	−0.1 (4)
N4—C3—C31—C32	56.1 (3)	C5—C51—C56—C55	−176.7 (2)
N4—C3—C31—C36	−123.2 (3)	C52—C51—C56—C55	−1.3 (4)
C2—C3—C31—C32	−66.8 (3)	C51—C52—C53—C54	1.3 (5)
C2—C3—C31—C36	113.9 (3)	C52—C53—C54—C55	−1.0 (5)
N4—C5—C6—N1	25.0 (3)	C53—C54—C55—C56	−0.4 (5)
N4—C5—C6—C61	−152.88 (19)	C54—C55—C56—C51	1.6 (4)
C51—C5—C6—N1	−151.0 (2)	C6—C61—C62—C63	−176.5 (2)
C51—C5—C6—C61	31.1 (3)	C66—C61—C62—C63	−1.4 (4)
N4—C5—C51—C52	−138.4 (2)	C6—C61—C66—C65	175.3 (2)
N4—C5—C51—C56	36.9 (3)	C62—C61—C66—C65	0.3 (4)
C6—C5—C51—C52	37.7 (3)	C61—C62—C63—C64	2.0 (4)
C6—C5—C51—C56	−147.0 (2)	C62—C63—C64—C65	−1.6 (4)
N1—C6—C61—C62	30.2 (3)	C63—C64—C65—C66	0.5 (4)
N1—C6—C61—C66	−144.8 (2)	C64—C65—C66—C61	0.1 (4)

Symmetry codes: (i) −*x*, −*y*, −*z*; (ii) −*x*+1, −*y*, −*z*; (iii) −*x*+1, −*y*+1, −*z*; (iv) −*x*, −*y*+1, −*z*; (v) −*x*, −*y*, −*z*−1; (vi) −*x*+1, −*y*+1, −*z*+1.

### Hydrogen-bond geometry (Å, °)

**Table:**

*D*—H···*A*	*D*—H	H···*A*	*D*···*A*	*D*—H···*A*
C65—H65···Cg(C31–C36)^iii^	0.93	2.97	3.834 (3)	156

Symmetry codes: (iii) −*x*+1, −*y*+1, −*z*.
